# Association between total ischemic time and in-hospital mortality after emergency PCI in patients with acute ST-segment elevation myocardial infarction: a retrospective study

**DOI:** 10.1186/s12872-022-02526-8

**Published:** 2022-03-04

**Authors:** Nan Gao, Xiaoyong Qi, Yi Dang, Yingxiao Li, Gang Wang, Xiao Liu, Ning Zhu, Jinguo Fu

**Affiliations:** 1 School of Graduate, Hebei Medical University, No. 361 Zhongshan East Street, Changan District, Shijiazhuang, Hebei Province 050000 People’s Republic of China; 2 Department of Cardiology, Baoding No. 1 Central Hospital, Baoding, 071000 Hebei Province People’s Republic of China; 3 Department of Cardiology, Hebei General Hospital, Shijiazhuang, 050000 Hebei Province People’s Republic of China; 4 Department of Cardiology, Cangzhou Central Hospital, Cangzhou, 061000 Hebei Province People’s Republic of China

**Keywords:** ST-elevated myocardial infarction, Symptom-to-balloon time, Ischemia time, Percutaneous coronary intervention

## Abstract

**Background:**

Symptom-to-balloon time (SBT) represents the total ischemic time in ST-elevated myocardial infarction (STEMI) and is associated with poor long-term outcomes. The study aimed to explore the association between SBT and in-hospital mortality after emergency percutaneous coronary intervention (PCI) in patients with acute STEMI.

**Methods:**

This retrospective, multicenter, observational study included patients admitted to the Hebei General Hospital, Baoding No. 1 Central Hospital, and Cangzhou Central Hospital from January 2016 to December 2018. The outcome was all-cause mortality during the hospital stay. Logistic regression models were established to explore the association between SBT and all-cause mortality during the hospital stay.

**Results:**

This study included 1169 patients: 876 males of 59.6 ± 11.4 years of age, and 293 females 66.3 ± 13.3 years of age. A first analysis showed EF had an interaction with SBT (P = 0.01). In patients with EF ≥ 50%, SBT was not an independent risk factor for postoperative all-cause mortality in the hospital (all P > 0.05). In patients with EF < 50%, SBT was an independent risk factor for postoperative all-cause mortality in the hospital [model 3: 1.51 (1.17, 1.54), P for trend = 0.01].

**Conclusions:**

SBT was independently associated with all-cause mortality in the hospital after PCI in patients with acute STEMI and EF < 50%. Specifically, the risk of in-hospital mortality for those with SBT ≥ 361 min is increased by 51% compared with those with SBT ≤ 120 min.

## Background

Acute myocardial infarction (AMI) is the leading cause of death in patients with cardiovascular diseases [1, 2]. AMI is responsible for about 1 million hospital admissions in the United States of America annually and 2 million in Europe [3]. The 30-day all-cause mortality is 7.3%-10.2% [4], and the 5-year all-cause mortality is 36.7% in patients with type 1 AMI, 62.5% for type 2, and 72.4% for myocardial injury [5]. Emergency primary percutaneous coronary intervention (PPCI) is considered as the first-line treatment for patients with acute ST-segment elevation myocardial infarction (STEMI) [6–8], and there is increasing evidence that emergency PCI can improve the outcomes of patients with AMI [9–12]. Still, many patients do not benefit from PCI, and the factors of poor prognosis include sex, thrombolysis in myocardial infarction (TIMI) classification, slow flow, infarct size, microvascular obstruction, intra-aortic balloon pump (IABP), use of β-blockers, use of angiotensin-converting enzyme inhibitors (ACEI)/angiotensin receptor blockers (ARB), symptom-to-door time (SDT), symptom-to-balloon time (SBT), ejection fraction (EF) [13–15].

Among the factors mentioned above, SDT represents the time during which the patient is without medical care and during which damage is uncontrolled, without specific treatments at all, and it is associated with the prognosis of PCI [14, 16]. On the other hand, the door-to-balloon time (DBT) represents when a patient is monitored and might receive medical treatments, but definitive treatment has not yet been undertaken; it is positively associated with the patient’s mortality [14, 16]. Differently, the SBT represents the entire myocardial ischemic period; it has no definite relationship with the short-term patient’s mortality [17, 18], but a recent study suggested a positive correlation between SBT and long-term mortality of patients with AMI after PCI [16]. Alsamara et al. [19] reported an association between SBT and decreased EF but no direct relationship with mortality. Hromadka et al. [20] showed that a longer ischemic time results more frequently in suboptimal TIMI score and worse EF. They also indicated that the factors for a worse prognosis were female sex, age, and obesity, which influenced the relationship between SBT and outcomes [20]. Thus, the association between SBT and the prognosis after PCI in patients with acute STEMI remains controversial and there is no consensus. It might vary among studies and populations.

Therefore, the present study aimed to explore the association between SBT and in-hospital mortality after emergency PCI in Chinese patients with acute STEMI.

## Methods

### Study design and patients

This retrospective, multicenter, observational study included patients admitted to the Hebei General Hospital, Baoding No. 1 Central Hospital, and Cangzhou Central Hospital from January 2016 to December 2018. The study was approved by the ethics committee of each participating hospital. Informed consent was obtained from the patients. All procedures performed in this study involving human participants were in accordance with the Declaration of Helsinki (as revised in 2013). The study was approved by institutional ethics research committee board of Baoding No. 1 Central Hospital (No. [2021]014).

The inclusion criteria were (1) meeting the diagnostic criteria for STEMI, and (2) underwent emergency PCI as per guidelines [7]. Patients with acute STEMI who underwent simple emergency coronary angiography but no PCI were excluded. According to a previous study, all eligible patients were assigned to four groups according to SBT: ≤ 120 min, 121–240 min, 241–360 min, and ≥ 361 min [21].

### Data collection and outcome

Patients’ data were collected, including general data, vital signs, auxiliary examination results, related data of chest pain, and data related to interventional therapy and medication. General data included age, sex, body mass index, history of smoking, and alcoholism. Past medical history included a history of hypertension, type 2 diabetes, myocardial infarction, and atrial fibrillation. Vital signs included pulse, systolic blood pressure, diastolic blood pressure, and Killip classification. Auxiliary examination results included white blood cell count (WBC), red blood cell count, hemoglobin, platelet count, serum potassium, sodium, chloride, creatinine, Acute kidney injury(AKI), uric acid, cholesterol, triglycerides, low-density lipoprotein cholesterol (LDL-C), high-density lipoprotein cholesterol (HDL-C), random blood glucose, creatine kinase (CK)-MB peak, and left ventricular EF (LVEF). Data of chest pain included the time from symptom onset to first medical contact and SBT. Data related to interventional therapy included the blood vessels involved by the lesion, lesion length, SYNTAX score, thrombus score, postoperative TIMI, blood flow grade, IABP, intraoperative slow blood flow, and intraoperative ventricular fibrillation. Medications after admission included β-blockers, ACEI/ARB, diuretic, and nicorandil.

The outcome of this study was in-hospital mortality, i.e., all-cause mortality during the hospital stay.

### Hemodynamic evaluation

The clinical evaluation was performed after the first medical contact upon admission. Killip I was defined as AMI patients without clinical symptoms of heart failure; Killip II as AMI complicated with left heart failure, and the moist rales of both lungs were less than 50% of the lung fields; Killip III as AMI complicated with acute pulmonary edema, and large, small, dry, and moist rales of the whole lung; Killip IV as AMI complicated with cardiogenic shock and other different degrees or stages of hemodynamic changes [22].

### Echocardiography evaluation

Transthoracic echocardiography was used to assess LVEF for the first time after the confirmation of STEMI. The patient was in the supine position. According to the frontier approaches of the American Society of Echocardiography, at least three consecutive cardiac cycles were used to measure the internal dimensions of the left ventricle (that is, the end-systolic dimension and end-diastolic dimension). The LVEF was calculated as LVEF (%) = [(LVEDD^3^-LVEDS^3^)/LVEDD^3^] × 100%.

### Evaluation of severity

The SYNTAX score is a scoring system designed based on the degree and extent of coronary lesions after coronary angiography [23].

The thrombus score was evaluated after the guidewire crossed the lesion (but before balloon dilation). The filling defect could be seen in multiple dimensions of angiography and was persistently present in multiple cardiac cycles. The inner membrane dissection caused by the guidewire in the false lumen was excluded. The thrombus score was graded as 0 = no thrombus; 1 = haziness; 2 = definite thrombus < 1/2 vessel diameter; 3 = definite thrombus ½ to 2 vessel diameters; 4 = definite thrombus > 2 vessel diameters; 5: unable to evaluate thrombus due to vascular occlusion [24].

TIMI blood flow classification was evaluated after PCI and non-compliant balloon dilation. TIMI flow grade was: grade 0 (no perfusion), i.e., no antegrade flow beyond the point of occlusion; grade 1 (penetration without perfusion), i.e., minimal antegrade flow after occlusion, but fails to opacify the entire distal coronary bed; grade 2 (partial perfusion), occlusion was complete but showed delayed perfusion of the distal coronary bed with contrast material; and grade 3 (complete perfusion) was antegrade flow to the entire distal bed at a normal rate [25].

AKI was defined as ≥ 26.52 μmol/L increase in serum creatinine within 48 h or a ≥ 50% increase in serum creatinine in 7 days [26].

### Statistical analysis

All data were analyzed using SPSS 21.0 (IBM, Armonk, NY, USA). The continuous data were tested for normal distribution using the Kolmogorov–Smirnov test. Normally distributed continuous variables are presented as means ± standard deviation and were analyzed using ANOVA and the LSD post hoc test. Non-normally distributed variables are presented as medians (interquartile ranges) and were analyzed using parametric tests. Categorical variables are presented using n (%) and were compared using the chi-square test. According to a previous study by the authors’ group, in addition to SBT, risk factors for mortality after emergency PCI in acute STEMI patients are sex, Killip grade, LM lesion, TIMI classification, symptom onset to first-medical-contact, Syntax score, WBC, CK-MB peak, use of β-blockers and ACEI/ARB after surgery, BMI, EF, and LDL [13]. AKI was an independent prognostic factor for long-term mortality among patients with STEMI complicated by cardiogenic shock (CS) and treated with primary percutaneous coronary intervention [26]. Therefore, the present study used in-hospital mortality as the dependent variable and SBT as the independent variable, and three logistic regression models were established to adjust for confounding factors to explore whether SBT was independently associated with the in-hospital mortality risk. Model 1: crude model. Model 2: adjusted for Killip, TIMI and AKI. Model 3: model 2 with sex, LM, SDT, SYNTAX, WBC, CK-MB, β-blocker, ACEI/ARB, BMI, and LDL-C. In addition, the product item was added to the regression model to detect whether there was an interaction between SBT and confounding factors. Two-sided P-values < 0.05 were considered statistically significant.

## Results

### Characteristics of patients

This study identified 1205 patients with acute STEMI, excluded 36 who did not undergo PCI, and finally included 1169 patients. Among them, 876 were males of 59.6 ± 11.4 years of age, and 293 were females 66.3 ± 13.3 years of age.

### Factor interaction with SBT

A first analysis showed EF had an interaction with the total ischemic time (P = 0.01), while all other factors had no interaction with SBT (all P > 0.17); then, a stratified analysis was conducted according to whether the LVEF was normal or not.

### Analysis of SBT in patients with EF < 50%

In patients with EF < 50% (Table 1), as SBT was prolonged, the proportion of patients with Killip IV, postoperative TIMI grade 0 and 1 and SDT increased; The proportion of patients with thrombus grade 4, receiving ACEI/ARB, the systolic and diastolic blood pressure levels were lower; The length of the coronary lesion was longer. Besides, there were statistical significance in the proportion of patients with Killip I, slow flow, receiving β-blocker and the length of the coronary lesion between groups, not keep pace with the prolonging of SBT (all P < 0.05) (Table 1).

**Table 1:** General characteristics of subjects with the number of EF < 50%

Characteristic	SBT (minutes)				*P*
	≤ 120	121–240	241–360	≥ 361	
	(n = 31)	(n = 130)	(n = 126)	(n = 92)	
Male, n (%)	25 (80.6)	101 (77.7)	98 (77.8)	70 (76.1)	0.96
Smoking history, n (%)	19 (61.3)	65 (50.0)	55 (43.7)	39 (42.4)	0.23
Drinking history, n (%)	12 (38.7)	33 (25.4)	34 (27.0)	7 (7.6)	0.5
DM history, n (%)	2 (6.5)	30 (23.1)	29 (23.0)	20 (21.7)	0.21
Hypertension history, n (%)	14 (45.2)	54 (41.5)	50 (39.7)	50 (54.3)	0.15
Myocardial infarction history, n (%)	1 (3.2)	12 (9.2)	13 (10.3)	8 (8.7)	0.67
Atrial fibrillation history, n (%)	1 (3.2)	2 (1.5)	3 (2.4)	3 (3.2)	0.85
*Killip classification, n (%)*					
I	23 (74.2)	100 (76.9)	102 (81.0)	59 (64.1)	0.04
II	7 (22.6)	23 (17.7)	11 (8.7)	15 (16.3)	0.1
III	1 (3.2)	3 (2.3)	6 (4.8)	4 (4.4)	0.74
IV	0 (0)	4 (3.1)	7 (5.5)	14 (15.2)	0.001
LM, n (%)	0 (0)	1 (0.8)	1 (0.8)	4 (4.3)	0.11
LAD, n (%)	20 (64.5)	77 (59.2)	70 (55.6)	47 (51.1)	0.5
LCX, n (%)	2 (6.5)	13 (10.0)	10 (7.9)	13 (14.1)	0.43
RCA, n (%)	9 (29.0)	39 (30.0)	45 (35.7)	30 (32.6)	0.77
*Grading of thrombus, n (%)*					
0	0 (0)	1 (0.8)	0 (0)	0 (0)	0.59
1	0 (0)	1 (0.8)	0 (0)	1 (1.1)	0.67
2	2 (6.5)	10 (7.7)	10 (7.9)	6 (6.5)	0.97
3	9 (29.0)	55 (42.3)	53 (42.1)	42 (45.7)	0.45
4	18 (58.1)	44 (33.1)	36 (28.6)	26 (28.3)	0.01
5	2 (6.4)	20 (15.3)	27 (21.4)	17 (18.4)	0.21
*TIMI classification, n (%)*					
0	0 (0)	0 (0)	1 (0.8)	5 (5.4)	0.008
1	0 (0)	0 (0)	0 (0)	3 (3.3)	0.02
2	1 (3.2)	13 (10.0)	10 (7.9)	11 (12.0)	0.47
3	30 (96.8)	117 (90.0)	116 (91.3)	73 (79.3)	0.13
Slow flow, n (%)	2 (6.5)	18 (13.8)	14 (11.1)	20 (21.7)	0.08
VF, n (%)	1 (3.2)	5 (3.8)	4 (3.2)	2 (2.2)	0.92
Application of IABP, n (%)	0 (0)	4 (3.1)	7 (5.6)	8 (8.7)	0.15
Administration of β-blocker, n (%)	22 (71.0)	98 (75.4)	81 (64.3)	50 (54.3)	0.01
ACEI/ARB, n (%)	19 (61.3)	74 (56.9)	59 (46.8)	35 (38.0)	0.02
Administration of diuretic, n (%)	10 (32.3)	32 (24.6)	35 (27.8)	27 (29.3)	0.79
Administration of nicorandil, n (%)	7 (22.6)	16 (12.3)	14 (11.1)	6 (6.5)	0.11
AKI, n(%)	3 (9.7)	8 (6.2)	7 (5.6)	15 (16.3)	0.04
Age, years, mean ± SD	60.06 ± 15.36	60.62 ± 11.44	60.14 ± 11.99	63.10 ± 11.01	0.28
BMI, kg/m^2^, mean ± SD	25.62 ± 3.89	25.57 ± 3.83	25.08 ± 3.34	25.41 ± 3.60	0.72
Symptom-to-door time, minutes, mean ± SD	34.94 ± 19.18	88.57 ± 39.34	137.52 ± 73.03	236.72 ± 155.35	0.001
P, bpm, mean ± SD	75.77 ± 17.81	76.44 ± 15.94	75.53 ± 14.97	75.68 ± 19.51	0.96
SBP, mmHg, mean ± SD	131.90 ± 23.90	131.43 ± 25.16	123.24 ± 23.61	119.67 ± 28.76	0.002
DBP, mmHg, mean ± SD	81.61 ± 14.31	81.34 ± 13.34	76.36 ± 14.48	73.25 ± 17.20	0.001
Length of lesion, mm, mean ± SD	24.90 ± 7.35	30.55 ± 10.98	28.59 ± 9.72	29.97 ± 11.62	0.04
Syntax score, mean ± SD	21.26 ± 6.60	22.27 ± 8.30	22.65 ± 8.06	23.94 ± 8.90	0.34
WBC, 10^9^/L, mean ± SD	10.83 ± 4.86	10.45 ± 3.04	11.07 ± 3.94	11.32 ± 3.40	0.32
RBC, 10^12^/L, mean ± SD	4.64 ± 0.55	4.52 ± 0.57	4.53 ± 0.65	4.48 ± 0.58	0.66
HGB, g/L, mean ± SD	144.85 ± 19.42	139.76 ± 17.61	141.30 ± 18.20	139.14 ± 18.20	0.43
PLT, 10^9^/L, mean ± SD	228.88 ± 53.25	218.05 ± 58.11	230.72 ± 62.61	229.07 ± 67.13	0.36
K, mmol/L, mean ± SD	4.03 ± 0.64	4.05 ± 0.47	3.99 ± 0.46	4.08 ± 0.50	0.56
Na, mmol/L, mean ± SD	138.48 ± 2.76	138.54 ± 3.38	138.74 ± 3.00	139.09 ± 4.15	0.67
Cl, mmol/L, mean ± SD	104.25 ± 2.37	102.29 ± 7.23	102.30 ± 3.34	101.44 ± 8.15	0.19
Cr, µmol/L, mean ± SD	83.60 ± 51.03	74.24 ± 20.31	74.83 ± 23.76	77.63 ± 29.77	0.33
UA, µmol/L, mean ± SD	335.20 ± 94.89	307.62 ± 87.65	312.18 ± 75.66	330.02 ± 105.64	0.17
CHOL, mmol/L, mean ± SD	4.54 ± 1.16	4.58 ± 0.97	4.61 ± 1.00	4.24 ± 1.00	0.05
TG, mmol/L, mean ± SD	1.44 ± 0.73	1.55 ± 1.17	1.65 ± 1.03	1.59 ± 0.79	0.71
HDL, mmol/L, mean ± SD	1.02 ± 0.22	1.04 ± 0.24	1.03 ± 0.37	1.01 ± 0.23	0.82
LDL, mmol/L, mean ± SD	3.03 ± 0.82	2.90 ± 0.73	2.93 ± 0.74	2.81 ± 0.69	0.45
CK-MB, ng/mL, mean ± SD	200.34 ± 108.59	185.11 ± 95.02	185.27 ± 98.24	201.82 ± 104.59	0.53
GLU, mmol/L, mean ± SD	7.56 ± 2.60	8.28 ± 3.29	8.73 ± 3.67	8.84 ± 3.86	0.25

Continuous variables are presented as mean ± standard deviation and categorical variables as %. ANOVA test (comparison of > 2 groups) for continuous and ordinal variables and the chi-squared test for categorical variables

SBT: symptom to balloon time; BMI: body mass index; DM: diabetes mellitus; LM: left main coronary artery; LAD: left anterior descending coronary artery; LCX: left circumflex branch coronary artery; RCA: right coronary artery; TIMI: thrombolysis in myocardial infarction; IABP: intra-aortic balloon pump, VF: ventricular fibrillation; ACEI: angiotensin-converting enzyme inhibitor, ARB: angiotensin receptor blocker, EF: ejection fraction, CK-MB: creatinine kinase MB; P: pulse; SBP: systolic blood pressure; DBP: diastolic blood pressure; WBC: white blood cell; RBC: red blood cell; HGB: hemoglobin; PLT: blood platelet; K: potassium; Na: sodium; Cl: chlorine; Cr: creatinine; UA: uric acid; CHOL: cholesterol; HDL: high-density lipoprotein cholesterol; LDL: low-density lipoprotein cholesterol; TG: triglycerides; GLU: glucose. *P* value < 0.05 was considered statistically significant

### Analysis of SBT in patients with EF ≥ 50%

In patients with EF ≥ 50% (Table 2), as SBT was prolonged, the proportion of patients with thrombus grade 5, SDT, was increased; the proportion of patients receiving β-blockers, ACEI/ARB, nicorandil was decreased; the SYNTAX score was higher. Besides, there were statistical significance in the proportion of patients with KillipI, II and IV, thrombus grade 2 and 4, TIMI blood flow of grade 0, 1 and 3, slow flow, IABP application, syntax score and the level of WBC, chlorine and glucose between groups, not keep pace with the prolonging of SBT (all P < 0.05) (Table 3).

**Table 2:** General characteristics of subjects with the number of EF ≥ 50%

Characteristic	SBT (minutes)				*P*
	≤ 120	121–240	241–360	≥ 361	
	(n = 90)	(n = 310)	(n = 272)	(n = 118)	
Male, n (%)	67 (74.4)	228 (73.5)	209 (76.8)	78 (66.1)	0.18
Smoking history, n (%)	50 (55.6)	147 (47.4)	132 (48.5)	46 (39.0)	0.12
Drinking history, n (%)	27 (30.0)	79 (25.5)	76 (27.9)	31 (26.3)	0.82
DM history, n (%)	16 (17.8)	68 (21.9)	45 (16.5)	30 (25.4)	0.16
Hypertension history, n (%)	44 (48.9)	165 (53.2)	127 (46.7)	63 (53.3)	0.39
Myocardial infarction history, n (%)	6 (6.7)	15 (4.8)	16 (5.9)	6 (5.1)	0.89
Atrial fibrillation history, n (%)	0 (0)	2 (0.6)	8 (2.9)	3 (2.5)	0.08
*Killip classification, n (%)*					
I	80 (88.9)	267 (86.1)	254 (93.4)	89 (75.4)	< 0.001
II	7 (7.8)	36 (11.6)	13 (4.8)	12 (10.2)	0.03
III	3 (3.3)	2 (0.6)	4 (1.5)	3 (2.5)	0.22
IV	0 (0)	5 (1.7)	1 (0.3)	14 (11.9)	< 0.001
LM, n (%)	0 (0)	2 (0.6)	1 (0.4)	1 (0.8)	0.81
LAD, n (%)	42 (46.7)	123 (39.7)	114 (41.9)	60 (50.8)	0.17
LCX, n (%)	10 (11.1)	51 (16.5)	29 (10.7)	11 (9.3)	0.09
RCA, n (%)	38 (42.2)	135 (43.5)	128 (47.1)	46 (39.0)	0.5
*Grading of thrombus, n (%)*					
0	2 (2.2)	2 (0.6)	1 (0.4)	0 (0)	0.2
1	1 (1.1)	7 (2.3)	3 (1.1)	2 (1.7)	0.71
2	5 (5.6)	22 (7.1)	36 (13.2)	7 (5.9)	0.02
3	31 (34.4)	119 (38.4)	119 (43.8)	51 (43.2)	0.32
4	47 (52.2)	121 (39.0)	66 (24.3)	35 (29.7)	< 0.001
5	4 (4.5)	39 (12.6)	47 (17.2)	23 (19.5)	0.006
*TIMI classification, n (%)*					
0	0 (0)	4 (1.3)	1 (0.4)	6 (5.1)	0.002
1	0 (0)	3 (1.0)	1 (0.4)	5 (4.2)	0.006
2	2 (2.2)	16 (5.2)	8 (2.9)	8 (6.8)	0.21
3	88 (97.8)	287 (92.5)	262 (96.3)	99 (83.9)	0.01
Slow flow, n (%)	3 (3.3)	30 (9.7)	18 (6.6)	22 (18.6)	0.001
VF, n (%)	6 (6.7)	10 (3.2)	10 (3.7)	5 (4.2)	0.52
Application of IABP, n (%)	0 (0)	5 (1.6)	2 (0.7)	6 (5.1)	0.009
Administration of β-blocker, n (%)	77 (85.6)	240 (77.4)	201 (73.9)	72 (61.0)	0.001
ACEI/ARB, n (%)	68 (75.6)	205 (66.1)	146 (53.7)	58 (49.2)	0.001
Administration of diuretic, n (%)	15 (16.7)	64 (20.6)	58 (21.3)	24 (20.3)	0.82
Administration of nicorandil, n (%)	24 (26.7)	52 (16.8)	19 (7.0)	6 (5.1)	0.001
AKI, n(%)	2 (2.2)	5 (1.6)	10 (3.7)	8 (6.7)	0.062
Age, years, mean ± SD	59.44 ± 11.13	59.77 ± 11.22	59.16 ± 12.03	61.37 ± 12.08	0.38
BMI, kg/m^2^, mean ± SD	25.09 ± 2.77	25.66 ± 3.22	25.44 ± 3.36	25.53 ± 2.77	0.5
Symptom-to-door time, minutes, mean ± SD	39.63 ± 23.39	82.12 ± 38.93	134.06 ± 69.88	181.08 ± 138.33	0.001
P, bpm, mean ± SD	75.98 ± 14.76	75.67 ± 14.18	74.87 ± 17.13	76.20 ± 16.65	0.85
SBP, mmHg, mean ± SD	131.54 ± 22.67	128.03 ± 22.95	129.65 ± 21.90	126.66 ± 24.55	0.38
DBP, mmHg, mean ± SD	81.53 ± 15.17	78.85 ± 14.53	81.03 ± 14.59	77.96 ± 14.72	0.09
Length of lesion, mm, mean ± SD	27.49 ± 11.41	28.70 ± 10.95	28.72 ± 10.36	29.09 ± 9.81	0.73
Syntax score, mean ± SD	20.15 ± 7.89	20.66 ± 7.83	20.24 ± 7.99	22.83 ± 9.09	0.03
WBC, 10^9^/L, mean ± SD	9.77 ± 2.76	10.77 ± 3.33	10.74 ± 3.60	11.37 ± 4.38	0.02
RBC, 10^12^/L, mean ± SD	4.50 ± 0.53	4.54 ± 0.61	4.49 ± 0.49	4.46 ± 0.56	0.64
HGB, g/L, mean ± SD	138.93 ± 17.51	138.48 ± 18.01	139.05 ± 15.69	138.97 ± 17.26	0.98
PLT, 10^9^/L, mean ± SD	229.69 ± 55.36	225.93 ± 58.73	229.64 ± 63.82	233.48 ± 82.61	0.73
K, mmol/L, mean ± SD	3.96 ± 0.40	3.94 ± 0.45	4.00 ± 0.55	4.06 ± 0.46	0.11
Na, mmol/L, mean ± SD	138.43 ± 3.85	138.87 ± 3.23	139.08 ± 2.96	138.86 ± 3.22	0.43
Cl, mmol/L, mean ± SD	104.14 ± 3.77	102.92 ± 3.61	102.55 ± 5.49	102.63 ± 3.31	0.02
Cr, µmol/L, mean ± SD	76.96 ± 20.62	72.82 ± 17.51	72.53 ± 22.30	72.85 ± 28.57	0.37
UA, µmol/L, mean ± SD	329.09 ± 89.98	313.02 ± 96.22	311.27 ± 84.37	308.79 ± 94.02	0.38
CHOL, mmol/L, mean ± SD	4.59 ± 0.97	4.59 ± 1.01	4.55 ± 0.98	4.50 ± 1.07	0.83
TG, mmol/L, mean ± SD	1.82 ± 1.11	1.66 ± 1.12	1.59 ± 0.91	1.78 ± 1.07	0.19
HDL, mmol/L, mean ± SD	1.02 ± 0.25	1.04 ± 0.25	1.03 ± 0.23	0.97 ± 0.20	0.06
LDL, mmol/L, mean ± SD	3.05 ± 0.81	2.98 ± 0.92	3.01 ± 0.88	2.87 ± 0.94	0.43
CK-MB, ng/mL, mean ± SD	163.33 ± 63.29	164.41 ± 74.37	166.03 ± 47.63	177.44 ± 89.39	0.31
GLU, mmol/L, mean ± SD	7.30 ± 3.09	8.28 ± 3.43	8.12 ± 3.14	8.75 ± 3.77	0.02

Continuous variables are presented as mean ± standard deviation and categorical variables as %. ANOVA test (comparison of > 2 groups) for continuous and ordinal variables and the chi-squared test for categorical variables

SBT: symptom to balloon time; BMI: body mass index; DM: diabetes mellitus; LM: left main coronary artery; LAD: left anterior descending coronary artery; LCX: left circumflex branch coronary artery; RCA: right coronary artery; TIMI: thrombolysis in myocardial infarction; IABP: intra-aortic balloon pump, VF: ventricular fibrillation; ACEI: angiotensin-converting enzyme inhibitor, ARB: angiotensin receptor blocker, EF: ejection fraction, CK-MB: creatinine kinase MB; P: pulse; SBP: systolic blood pressure; DBP: diastolic blood pressure; WBC: white blood cell; RBC: red blood cell; HGB: hemoglobin; PLT: blood platelet; K: potassium; Na: sodium; Cl: chlorine; Cr: creatinine; UA: uric acid; CHOL: cholesterol; HDL: high-density lipoprotein cholesterol; LDL: low-density lipoprotein cholesterol; TG: triglycerides; GLU: glucose. P-value < 0.05 was considered statistically significant

**Table 3:** Adjusted odds ratios (with 95% CIs) from logistic regression models for associations of SBT with death

Models	SBT (min)				P for trend
	≤ 120	121–240	241–360	≥ 361	
*EF < 50%*					
No. of subjects, n	31	130	126	92	
Death cases, n	0	3	9	42	
Model 1	1	1.02 (0.91, 1.14)	1.03 (1.01, 1.13)	1.23 (1.08, 1.38)	< 0.001
Model 2	1	1.05 (0.89, 1.16)	1.10 (1.02, 1.15)	1.15 (1.00, 1.47)	0.15
Model 3	1	1.03 (1.01, 1.18)	1.17 (1.03, 1.34)	1.51 (1.17, 1.54)	0.01
*EF ≥ 50%*					
No. of subjects, n	90	310	272	118	
Death cases, n	0	8	3	20	
Model 1	1	1.05 (0.89,1.28)	1.15 (1.04, 1.46)	1.34 (1.12, 1.59)	< 0.001
Model 2	1	1.04 (0.81, 1.17)	1.07 (0.82, 1.54)	1.13 (1.01, 1.73)	0.09
Model 3	1	1.01 (0.85, 1.24)	1.18 (1.01, 2.41)	1.11 (1.04, 1.94)	0.08

Model 1: Crude model. Model 2: Adjusted for Killip, TIMI and AKI. Model 3: Additionally adjusted for sex, LM, symptom-to-door time, syntax, WBC, CK-MB, β-blocker, ACEI/ARB, BMI, and LDL. P-value < 0.05 was considered statistically significant.

### Multivariable analyses

In patients with EF ≥ 50%, SBT was not an independent risk factor for postoperative all-cause mortality in the hospital (model 2: P = 0.09; model 3: P for trend = 0.08). In patients with EF < 50%, SBT was an independent risk factor for postoperative all-cause mortality in the hospital [model 3: 1.51 (1.17, 1.54), P for trend = 0.01] (Table 3).

## Discussion

The association between SBT and the prognosis after PCI in patients with acute STEMI remains controversial. The results of this study suggest that SBT is an independent risk factor for in-hospital all-cause mortality after emergency PCI in acute STEMI patients with EF < 50%. The risk of in-hospital mortality for patients with SBT ≥ 361 min was increased by 51% compared with SBT ≤ 120 min. However, there was no correlation between SBT and in-hospital mortality in patients with EF ≥ 50%. This study refines the previous studies by showing that the association of SBT with short-term mortality is only significant in patients with impaired EF, while no significant association was seen in patients with normal EF.

In Model 2, AKI, Killip grade and TIMI grade were included in the model to adjust the influence of these three factors on the outcome. The results showed that after adjusting the above three factors, whether EF > 50% group or EF < 50% group, the trend P values were all greater than 0.05, suggesting that there was no statistical relationship between SBT and in-hospital mortality risk. These results suggest that AKI, Killip and TIMI grade are important risk factors for in-hospital mortality in patients with ST-segment elevation myocardial infarction. This is similar to the results of Hayıroğlu Mİ et al. and Çinar T et al. [26, 27].

Prasad et al. [28] used the myocardial blush grade (MBG) and resolution of ST-segment elevation (STR) to determine the degree of myocardial injury after AMI-related infarct coronary recanalization, and the results showed that the degree of myocardial injury was aggravated with the prolongation of SBT, which was stronger than the correlation between DBT and myocardial injury. Shiomi et al. [18] also found that AMI patients with a short SBT (< 3 h) had a lower risk of mortality and heart failure than those with a long SBT (> 3 h), but patients with DBT > 90 min and those with DBT < 90 min had a similar risk of mortality and heart failure; only when SBT < 120 min, the risk of mortality and heart failure in AMI patients with DBT < 90 min was reduced. It suggested that SBT can better reflect the degree of myocardial injury and necrosis than DBT, thereby affecting the outcomes of patients, as suggested by more recent studies [14, 16, 20]. Like these studies, the present study revealed that with the prolongation of SBT, the risk of in-hospital mortality in AMI patients with EF < 50% was increased, but there was no increase in the risk of mortality in AMI patients with EF ≥ 50%, which has not been observed in previous studies. As is known, myocardial cells begin to be irreversibly injured after 20 min of coronary occlusion, and myocardial cells will be completely necrotic by about 6 h [29]. The whole progression of the infarct depends upon the presence or absence of the establishment of collateral circulation, ischemic preconditioning, and SBT. EF < 50% at admission suggests the presence of a larger area of myocardial cell injury and necrosis, and the infarct-related coronary arteries are still occluded or have no collateral circulation. Therefore, with the prolongation of SBT, the number of necrotic myocardial cells continues to increase, and in-hospital mortality will be further increased. On the other hand, for patients with EF ≥ 50%, the number of damaged and necrotic myocardial cells is still small, which might be due to the presence of collateral circulation or infarct-related coronary occlusion, recanalization, and reocclusion, myocardial cells underwent the process of ischemic preconditioning, reducing the number of necrotic myocardial cells, so these patients did not show an increase in the in-hospital mortality with the prolongation of SBT. Nevertheless, the patients with EF < 50% might include some patients with early-stage heart failure due to any cause, and such patients would have a poorer prognosis. Since these patients are usually undiagnosed because of no particular or non-specific symptoms, the present study cannot answer this point.

Unlike the present study, previous findings suggest that DBT is associated with the short- and long-term mortality of AMI patients undergoing emergency PCI, while SBT has no obvious correlation with the mortality of patients. A clinical observation study of 43,801 AMI patients by Rathore et al. [30]found that longer DBT was associated with a higher risk of in-hospital mortality. Based on data from the Second National Registry of Myocardial Infarction, studies by Cannon et al. [17]showed that the average DBT recorded by the center was 116 min. As the DBT prolonged, the mortality was increased, while there was no correlation between the SBT and mortality. McNamara et al. [31]obtained similar findings. The mortality of AMI patients was associated with DBT but did not correlate with SBT. That may be due to various confounding factors like the levels of the hospitals and the general characteristics of the patients. In addition, pooling all the patients together without stratification based on EF might also dilute the significance of the relationships. Still, the results of the present study need to be confirmed.

### Limitations and prospect

This study was a retrospective study, not a prospective, randomized, controlled clinical trial. Although the patients have been selected from multiple centers, selection bias is inevitable. Previous studies have shown that IABP use was an independent predictor of in-hospital mortality risk [32]. But this study did not confirm this result. This may be due to the low number of IABP applications. Of the 1169 patients with acute ST-segment elevation myocardial infarction, 32(2.7%) patients received IABP, which was related to the size of the hospital and the level of medical care, the next step is to expand the number of treatment centers and increase the sample size. Further research with larger-scale clinical case selection might be closer to the real world, achieving more convincing results. Secondly, research at the cellular level can further explain the findings of the present study, which will be our next research direction.

## Conclusions

In acute STEMI patients undergoing emergency PCI with EF < 50%, the risk of in-hospital mortality for those with total ischemic time ≥ 361 min is increased by 51% compared with those with a total ischemic time of ≤ 120 min. There is no association between total ischemic time and in-hospital mortality in patients with EF ≥ 50%. Thus, for AMI patients with EF < 50% upon admission, it is imperative to shorten SBT as much as possible to reduce mortality and improve prognosis.

## Data Availability

All data generated or analyzed during this study are included in this published article.
